# Analysis of Engineered Tobacco Mosaic Virus and Potato Virus X Nanoparticles as Carriers for Biocatalysts

**DOI:** 10.3389/fpls.2021.710869

**Published:** 2021-08-06

**Authors:** Juliane Schuphan, Ulrich Commandeur

**Affiliations:** Institute for Molecular Biotechnology, Rheinisch-Westfälische Technische Hochschule (RWTH) Aachen University, Aachen, Germany

**Keywords:** cellulase, covalent immobilization, plant virus nanoparticles, tobacco mosaic virus, SpyTag/SpyCatcher

## Abstract

Plant virus nanoparticles are promising candidates for the development of novel materials, including nanocomposites and scaffolds/carriers for functional molecules such as enzymes. Their advantages for enzyme immobilization include a modular organization, a robust and programmable structure, and a simple, cost-effective production. However, the activity of many enzymes relies on posttranslational modification and most plant viruses replicate in the cytoplasm, so functional enzymes cannot be displayed on the virus surface by direct coat protein fusions. An alternative display system to present the *Trichoderma reesei* endoglucanase Cel12A on potato virus X (PVX) using SpyTag/SpyCatcher (ST/SC) technology was recently developed by the authors, which allows the carrier and enzyme to be produced separately before isopeptide conjugation. Although kinetic analysis clearly indicated efficient biocatalyst activity, the PVX carrier interfered with substrate binding. To overcome this, the suitability of tobacco mosaic virus (TMV) was tested, which can also accommodate a larger number of ST peptides. We produced TMV particles displaying ST as a new platform for the immobilization of enzymes such as Cel12A, and compared its performance to the established PVX-ST platform in terms of catalytic efficiency. Although more enzyme molecules were immobilized on the TMV-ST particles, we found that the rigid scaffold and helical spacing significantly affected enzyme activity.

## Introduction

The development of efficient and sustainable bioprocesses often involves the immobilization of biocatalysts, replacing traditional chemical conversion methods (Hasan et al., 2006). Enzymes are not only specific and selective for certain substrates, but also promote environmental sustainability by reducing the consumption of chemicals and avoiding the production of toxic by-products. However, industrial processes based on enzymes must overcome the low stability and reusability of soluble enzymes and the high operational costs, which can be addressed by immobilization technologies (Betancor and Luckarift, 2008; Husain et al., 2011; Srivastava and Singh, 2013; Zhang et al., 2015; Singh et al., 2017). Enzyme performance can also be improved by using biotechnology to optimize characteristics such as activity, thermostability, or salt tolerance (Gilmore et al., 2015; Behera and Ray, 2016; Bischof et al., 2016). This strategy has led to the development of

artificial multienzyme complexes, known as designer cellulosomes, that convert lignocellulosic plant biomass into fermentable sugars for biofuel production (Bayer et al., 1994; Fierobe et al., 2001, 2002, 2005; Arfi et al., 2014; Stern et al., 2015). Cellulase efficiency can be increased further by using scaffold materials with a high surface area to volume ratio, maximizing the loading of synergistically acting enzymes. Such scaffold materials must ensure the sufficient diffusion of substrates and products, providing a microenvironment that enhances enzymatic performance (Misson et al., 2015).

The functionalization of plant virus nanoparticles (VNPs) has applications in diverse areas such as medicine (vaccination, drug delivery, and biological imaging), electronics, and tissue engineering, benefitting from the well-characterized genome sequence and modular organization of viruses (Butler, 1984; Wen and Steinmetz, 2016; Röder et al., 2019; Grinzato et al., 2020). The virus capsid is composed of multiple identical coat protein (CP) subunits, which are easily modified by the chemical conjugation of exposed functional groups and/or genetic engineering to selectively attach molecules and fuse peptides conferring new functions (Pokorski and Steinmetz, 2011; Bruckman and Steinmetz, 2014; Lee et al., 2014; Wen and Steinmetz, 2016; Röder et al., 2019). However, the coupling efficiency of chemical conjugation is often low (21–86%), depending on the target molecule size and coupling strategy (Schlick et al., 2005; Holder et al., 2010; Venter et al., 2011; Wen et al., 2012). Furthermore, genetic engineering strategies are hindered by the natural tendency of plant viruses to select for small genomes by eliminating unnecessary sequences (including large transgenes) during infection, although this can be addressed to some extent by using optimized viral vectors or the ribosomal skip (overcoat) strategy (Cruz et al., 1996; Dickmeis et al., 2014; Röder et al., 2017a). Another challenge is that the isoelectric point (pI) and amino acid composition of CPs must remain within certain limits to permit assembly, which restricts the nature of peptides that can be displayed on the surface (Beachy et al., 1996; Bendahmane et al., 1999; Lico et al., 2006; Betti et al., 2012). Finally, post-translational modifications are important for many enzymes and essential for most cellulases, but this is not possible with engineered VNPs due to their replication and assembly in the cytoplasm.

Tobacco mosaic virus (TMV), the type member of the genus *Tobamovirus* (*Virgaviridae*), is one of the best characterized plant viruses and a popular choice for nanotechnology applications (Lee et al., 2020). The 6.4 kb RNA genome is encapsidated by 2,130 helically assembled CP subunits to form a rigid rod-shaped 300 ×18 nm particle with a 4 nm inner channel, which has been characterized by X-ray fiber diffraction to 2.9 Å resolution (Zimmern, 1977; Belkum et al., 1985; Namba et al., 1989; Klug, 1999). To display peptides on the outer particle surface, coding sequences can be inserted at the 5′ or 3′ end of the *cp* gene, between amino acid positions 63 and 66, or close to the C-terminus between positions 154 and 155, using the overcoat strategy or a leaky stop codon (Bloomer et al., 1978; Skuzeski et al., 1991; Turpen et al., 1995; Röder et al., 2017a). Recently, the authors developed an alternative display platform for the flexible rod-shaped potato virus X (PVX) using the SpyTag/SpyCatcher (ST/SC) covalent protein-based tag system (Zakeri et al., 2012; Röder et al., 2017b). This system was engineered by splitting the CnaB2 domain of the fibronectin-binding protein from *Streptococcus pyogenes* into the small ST (13 residues) and SC (116 residues), which spontaneously form an irreversible isopeptide bond (Zakeri et al., 2012). Although a recent development, this system has been used for many different approaches and has been shown to be a versatile tool for plant metabolic engineering (Hatlem et al., 2019; Keeble and Howarth, 2020; Lang et al., 2020). The PVX CP was modified to carry the ST peptide and successful conjugation was demonstrated by the irreversible and specific binding of *Trichoderma reesei* endoglucanase Cel12A fused to SC leading to a coupling efficiency of ~67% and higher catalytic efficiency. However, the comparative kinetic analysis of free and immobilized Cel12A-SC indicated that PVX interferes with substrate binding. The authors therefore attempted to circumvent this limitation using the more rigid TMV scaffold, which can also accommodate ~60% more ST peptides. The objective was to produce TMV particles displaying ST as new platform for the immobilization of proteins with essential posttranslational modifications, in particular Cel12A. The catalytic efficiency of the new TMV-ST particles to the existing PVX-ST platform was compared.

## Materials and Methods

### Vector Construction

Sequences for ST, the flexible glycine-serine linker (G_4_S)_3_ and the pI-adjusting motif DEADDAED were fused to the 3′ end of the TMV *cp* gene by PCR using *Pfu* DNA Polymerase (Promega, Mannheim, Germany) and specific primers PacI-CP (5′-AAA TTA ATT AAA TGC CTT ATA CAA TCA ACT CTC-3′) and G4S-DD-ST-NotI (5′-AAA GCG GCC GCT TAC TTA GTA GGC TTA TAA GCA TCA ACC ATA ACA ATA TGA GCC ATA TCT TCT GCG TCA TCA GCC TCG TCG CTA CCG CCT CCA CCA CTC C-3′), which also introduced the named restriction sites. GoTaq G2 DNA Polymerase (Promega) was used for the 3′ adenylation of the PCR product, before transfer to vector pCR2.1 TOPO (Thermo Fisher Scientific, Dreieich, Germany) and the transformation of chemically competent TOP10 cells according to the recommendations of the manufacturer. The integrity of the product was confirmed by PCR using primers M13-fw (5′-GTT GTA AAA CGA CGG CCA GT-3') and M13-rev (5'-ACA CAG GAA ACA GCT ATG AC-3′). After digestion with PacI and NotI-HF (New England Biolabs, Frankfurt am Main, Germany), the *cp-g4s-dd-st* sequence was inserted into the pTRBOc vector (derivative of pTRBO (Lindbo, 2007), Dr. C. Dickmeis, unpublished data), which had been linearized with the same restriction enzymes and dephosphorylated with calf intestinal alkaline phosphatase (New England Biolabs) to prevent auto-ligation. Ligation was carried out at 16°C overnight using T4 DNA ligase (Promega) before precipitation with glycogen/ethanol and electroporation of *Escherichia coli* DH5α cells. Transformation was confirmed by colony PCR with primers TMV5482f (5′-TTG ATG AGT TCA TGG AAG-3′) and TMV6269r (5′-TTC GAT TTA AGT GGA GGG-3'), and the resulting plasmid pTMV-ST was verified by sequencing *cp-g4s-dd-st* (Eurofins Genomics, Ebersberg, Germany). Vector pTRAkt-Cel12A-SC was used for the transformation of *Agrobacterium tumefaciens* GV3101::pMP90RK cells as previously described (Röder et al., 2017b). The vector sequences can be found in the Supplementary Material.

### Plant Inoculation, Infiltration, and Maintenance of *Nicotiana benthamiana*

For inoculation, the leaf surface of 4-week-old *Nicotiana benthamiana* plants was gently abraded with Celite 545 and 7–10 μg of pTMV-ST. After incubation for 20–30 min, the leaves were rinsed with water to remove excess DNA and Celite. We also used 10 μg of wild-type TMV particles (strain U1) to inoculate *N. benthamiana* plants as described above. For infiltration, *Agrobacterium tumefaciens* was cultivated in yeast extract broth (YEB) medium (0.1% (w/v) yeast extract, 0.5% (w/v) tryptone, 0.5% (w/v) beef extract, 0.5% (w/v) sucrose, 2 mM MgSO_4_) supplemented with 100 μg ml^−1^ carbenicillin, 50 μg ml^−1^ kanamycin, and 50 μg ml^−1^ rifampicin at 26°C for 3 d, shaking at 160 rpm. The medium with 10 μM acetosyringone, 10 mM MES, (pH 5.6), and 10 mM glucose was then supplemented and incubated for a further 16–20 h. The OD_600_ was then adjusted to 1.0 with 2 × infiltration medium (10% (w/v) sucrose, 0.36% (w/v) glucose, 0.86% (w/v) MS salts, pH 5.6) and mixed with *A. tumefaciens* carrying the pTRAkt-p19 silencing suppressor (the OD_600_ was adjusted to 0.2). The suspension was then supplemented with 200 μM acetosyringone and incubated at room temperature for 1 h. A syringe without a needle was used to infiltrate the leaves. The plants were cultivated in a phytochamber (12 h photoperiod at 5,000–10,000 lux, 26/20°C day/night, 70% humidity). Plants were harvested 12–18 days post-inoculation (dpi) depending on the progress of the infection, and infiltrated leaves were collected 6 days post-infiltration.

### Extraction and Purification of TMV Particles

Proteins were co-extracted from leaves systemically infected with pTMV-ST and infiltrated with pTRAkt-Cel12A-SC by homogenization of the leaf mixture in two volumes of phosphate buffered saline (PBS) (137 mM NaCl, 2.7 mM KCl, 10.1 mM Na_2_HPO_4_, 1.5 mM KH_2_PO_4_, pH 7.4) supplemented with 10 mM Na_2_S_2_O_5_. The homogenate was incubated for 30 min at 4°C, and cell debris was removed by centrifugation (20,000 × g for 15 min at 4°C). TMV-ST particles with covalently attached Cel12A were purified by layered-bead chromatography using HiScreen Capto Core700 resin on an Äkta pure25 system (GE Healthcare Life Sciences, Chicago, IL, USA), and were concentrated as previously described (Röder et al., 2017b). Purified wild-type TMV particles were obtained as described in the protocol of the International Potato Centre (Lima, Peru) with modifications (Uhde-Holzem et al., 2010). Briefly, the sucrose cushion centrifugation step was omitted and the pellet was directly resuspended after clarification. The subsequent centrifugation step (7,800 × g, 10 min, 4°C) differed from the original protocol (2,000 × g, 5 min, 4°C), and the second ultracentrifugation step to sediment the virions lasted up to 4 h instead of 1 h. The final clarification step was also omitted.

### Extraction and Purification of Endoglucanases

Infiltrated leaves expressing Cel12A (Dr. H. Klose, unpublished data) or Cel12A-SC were harvested and homogenized in two volumes of PBS. To remove cell debris, the homogenate was centrifuged at 20,000 × g for 15 min at 4°C. Extracted recombinant proteins contained a His_6_ tag and were therefore purified by gravity flow immobilized metal ion affinity chromatography over nickel-nitrilotriacetic acid (Ni-NTA) agarose (Qiagen, Hilden, Germany) according to the manufacturer's recommendations. PBS was used for equilibration and washing. For elution, PBS was supplemented with 250 mM imidazole.

### Deglycosylation

Purified Cel12A-SC was deglycosylated using endoglycosidase H (Endo H_f_, New England Biolabs) according to the recommendations of the manufacturer.

### Protein Detection

Samples (15 μl plant extracts or 2.5 μg purified TMV-ST/Cel12A-SC) were boiled for 5 min in 5 × reducing loading buffer before separation by sodium dodecyl sulfate polyacrylamide gel electrophoresis (SDS-PAGE) on a 12% resolving gel and 4% stacking gel (Laemmli, 1970). P7712 or P7719 Prestained Protein Standards (New England Biolabs) were used for sizing. The separated proteins were either stained with Coomassie Brilliant Blue or transferred to a Hybond-C nitrocellulose membrane (GE Healthcare Life Sciences) using a semidry blotting system (BioRad Laboratories, Munich, Germany) for western blot analysis. The membrane was blocked for at least 45 min in 4% (w/v) skimmed milk in PBS, and target proteins were detected by incubation at room temperature overnight with a primary polyclonal anti-tobacco mild green mosaic virus (TMGMV) antibody, described in the text as an anti-TMV antibody (DSMZ, Braunschweig, Germany), or a monoclonal anti-His antibody (Qiagen) diluted 1:5000 in PBS, followed by incubation for at least 3 h with an alkaline phosphatase (AP)-labeled goat-anti rabbit (GAR^AP^) or goat-anti mouse (GAM^AP^) secondary antibody (Dianova, Hamburg, Germany) diluted 1:5000 in PBS. The signal was visualized by staining with nitroblue tetrazolium chloride/5-bromo-4-chloro-3-indolyphosphate *p*-toluidine salt (Carl Roth, Karlsruhe, Germany). Coupling efficiency was determined by the densitometric analysis of target bands in western blots using ImageJ v1.50f as previously described (Röder et al., 2017b).

### Protein Quantification

The concentrations of purified proteins, wild-type TMV, and TMV-ST/Cel12A-SC complexes were determined using a Bradford assay containing RotiQuant reagent (Carl Roth) according to the recommendations of the manufacturer. Three replicates were taken at each measurement point. Bovine serum albumin (BSA) was used as the calibration standard, and PBS as the blank.

### Analysis of Viral RNA

Total RNA was extracted from systemically infected *N. benthamiana* leaves using the RNeasy Plant Mini Kit (Qiagen) according to the instructions of the manufacturer. RNA samples (3 μg) were treated with 3 U DNase I (Thermo Fisher Scientific) prior to the reverse transcription of viral genomic RNA using Moloney Murine Leukemia Virus (M-MLV) Reverse Transcriptase RNase H Minus Point Mutant (Promega). DNase-digested viral RNA (1 μg) was mixed with 0.5 μl TMV6269r primer and incubated for 10 min at 80°C and for 10 min at 4°C to ensure primer annealing. For cDNA synthesis, we mixed 5 μl 5 × M-MLV reaction buffer, 1 mM dNTPs, 2.5 μl DEPC-treated water, and 1 μl M-MLV reverse transcriptase and incubated the reaction for 30 min at 40°C, 20 min at 45°C, 20 min at 50°C, 20 min at 55°C, and 20 min at 70°C. The integrity of TMV-ST was determined by PCR using TMV-specific primers TMV5482f and TMV6269r.

### Visualization of TMV Particles Displaying Cel12A

Immunosorbent electron microscopy was used to visualize complex formation in plant extracts. Pioloform-coated nickel grids (Plano, Wetzlar, Germany) were floated on 40 μl anti-TMV or anti-His antibodies (diluted 1:100 in PBS) for 20–30 min at room temperature. The grids were washed with PBST (PBS plus 0.5% (v/v) Tween-20), and blocked with 40 μl 0.5% (w/v) BSA (British BioCell, Cardiff, UK) for another 20–30 min and washed again. Then, 40 μl of leaf extracts were added and incubated for 30–60 min before washing with PBST. VNPs were detected with primary anti-His or anti-TMV antibodies diluted 1:1000 in PBS and incubation overnight. Secondary 15 nm gold-conjugated GAM or GAR antibodies (British BioCell, diluted 1:100 in PBS) were used to decorate the particles by incubation for 2–4 h. After extensively washing with PBST, PBS, and deionized water, particles were contrasted with 1% (w/v) uranyl acetate. The grids were air-dried and analyzed by transmission electron microscopy using a Zeiss EM10 microscope (Carl Zeiss, Jena, Germany). The thermostability of purified TMV-ST/Cel12A nanoparticles was determined by heating for another 1 h at 50°C before labeling with the immunogold antibody pairs described above.

### Enzymatic Assays

Zymography was used to visualize the enzymatic activity of 5 μl leaf extracts or 2.5 μg purified TMV-ST/Cel12A-SC particles separated by SDS-PAGE (without sample boiling) in gels containing 0.15% (w/v) carboxymethylcellulose (CMC) (Sigma-Aldrich, Munich, Germany). The gel was washed twice with renaturation buffer (50 mM sodium acetate, 20% (v/v) propan-2-ol, pH 4.8) for 30 min at room temperature, followed by three washes in 50 mM sodium acetate (pH 4.8) for 15 min each. The enzymatic reaction was incubated in the same buffer for 15 min at 50°C and was stopped by reducing the temperature to 4°C and shifting the pH (50 mM Tris-HCl, pH 7.5) for 30 min. The gel was stained with 0.1% (w/v) Congo Red (Sigma-Aldrich) for 30 min, destained in 1 M NaCl, and briefly incubated in 0.3% (v/v) acidic acid to increase the contrast. The Onozuka R-10 multicomponent enzyme system from *T. viridae* was used as a control. Statistical significance was calculated using an unpaired two-tailed *t*-test in Microsoft Excel 2019.

Nondenaturing colorimetric or fluorometric assays were used to quantify enzyme activity or determine kinetic parameters, respectively. Briefly, thermotolerance (20–80°C) and pH tolerance (pH 3.0–8.0) of purified TMV-ST/Cel12A-SC nanoparticles were estimated by Azo-CMC analysis at a protein concentration of 0.5 μM as previously described (Röder et al., 2017b) using purified TMV-ST particles or free enzymes (Cel12A, Cel12A-SC) as controls. PBS served as blank. Three replicates were taken at each measurement point. Enzyme kinetics were calculated using the substrate 4-methylumbelliferyl-β-d-cellobioside (4-MUC) (Sigma-Aldrich) as previously described (Röder et al., 2017b). The protein concentration of the same samples was set to 0.75 μM and incubated at optimal temperature and pH (as investigated in Azo-CMC assay before). The conversion rate was calculated against 4 MU standards. Kinetic constants were calculated by fitting the reaction rate data to the Michaelis-Menten equation using GraphPad Prism.

To extrapolate kinetics for a 100% enzyme coverage of PVX or TMV, the coupling efficiency as calculated by densitometric analyses of target bands in western blots was used. The total protein content has been adjusted accordingly. Estimated kinetics for a 100% coverage were then calculated using GraphPad Prism based on the empirically measured activities but with the lower enzyme concentration.

### Software

Clone Manager Professional Suite 8 was used to calculate the molecular weights and pIs of the proteins. Molecular graphics were prepared using the UCSF Chimera package developed by the Resource for Biocomputing, Visualization, and Informatics at the University of California, San Francisco (NIGMS P41-GM103311). PDB ID: SpyTag/SpyCatcher-−4MLS, Cel12A-−1H8V, TMV CP-−3J06. Glycosylation sites were predicted using the NetNGlyc 1.0 server (Technical University of Denmark). Charts were designed using Microsoft Office Excel 2019 (Microsoft, Redmond, WA, USA). Enzyme kinetics were plotted in GraphPad Prism v7.03 (GraphPad Software, La Jolla, CA, USA).

## Results

**Figure 1:**
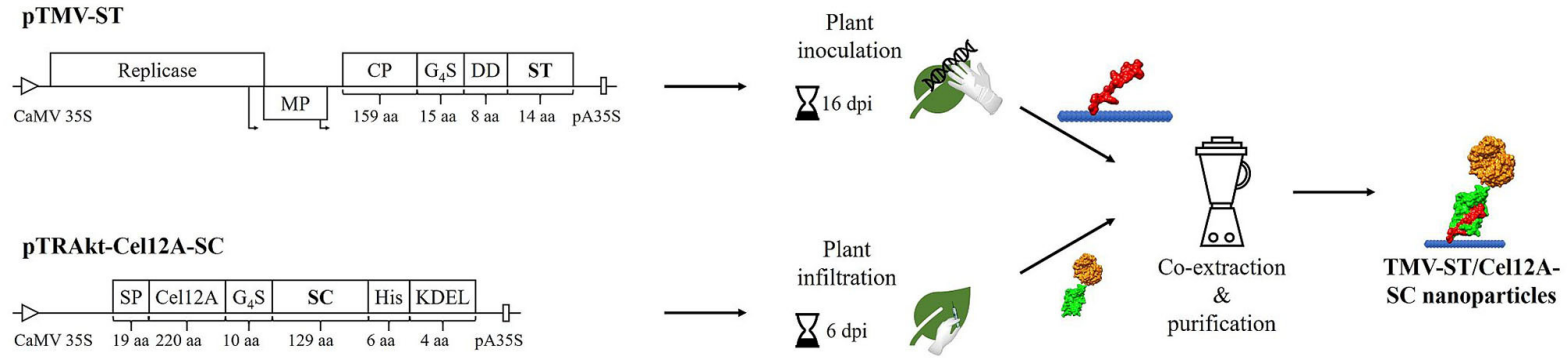
Schematic representation of the production of TMV-ST/Cel12A-SC nanoparticles. The SpyTag (ST) gene was directly fused to the 3′ end of the TMV coat protein (CP) gene with additional sequences encoding a flexible linker (G_4_S) and a pI-adjusting peptide (DD). The resulting viral vector was used to inoculate *N. benthamiana* plants, causing systemic infection and producing recombinant particles. *T. reesei* Cel12A endoglucanase was fused *via* a linker to the 5′ end of the SpyCatcher (SC) sequence, and was used for agroinfiltration, producing monomeric fusion proteins. ST and SC formed an isopeptide bond during the co-extraction and purification of particles. CaMV 35S/pA35S, Cauliflower mosaic virus 35S promoter/polyadenylation sequence; Cel12A, *T. reesei* endoglucanase Cel12A; DD, pI-adjusting sequence encoding the peptide DEADDAED; dpi, days post-infection/infiltration; G_4_S, protein linker; His, His_6_ tag; KDEL, endoplasmic reticulum retention signal; MP, movement protein; Non-infected, non-infected *N. benthamiana* plant extract; Replicase, TMV replicase; SC, SpyCatcher; SP, codon-optimized murine signal peptide from mAb24; ST, SpyTag; Arrows, subgenomic promoter-like sequences.

The ST sequence was inserted at the 3′ end of the TMV *cp* gene with an additional flexible linker and a pI-adjusting sequence (DD). The resulting recombinant viral vector was used to infect *N. benthamiana* plants by mechanical inoculation (Figure 1). The corresponding SC sequence was fused to *T. reesei* endoglucanase Cel12A in our previous study and transiently expressed in *N. benthamiana* by agroinfiltration (Röder et al., 2017b). The covalent binding of ST to SC was enabled during the homogenization of systemically infected leaves containing TMV-ST nanoparticles and infiltrated leaves containing Cel12A-SC, and the conjugated particles were subsequently purified by layered-bead chromatography. It should be mentioned here that TMV-ST particles achieve a yield that is about 5-7 times lower than that of TMV wild-type particles.

**Figure 2:**
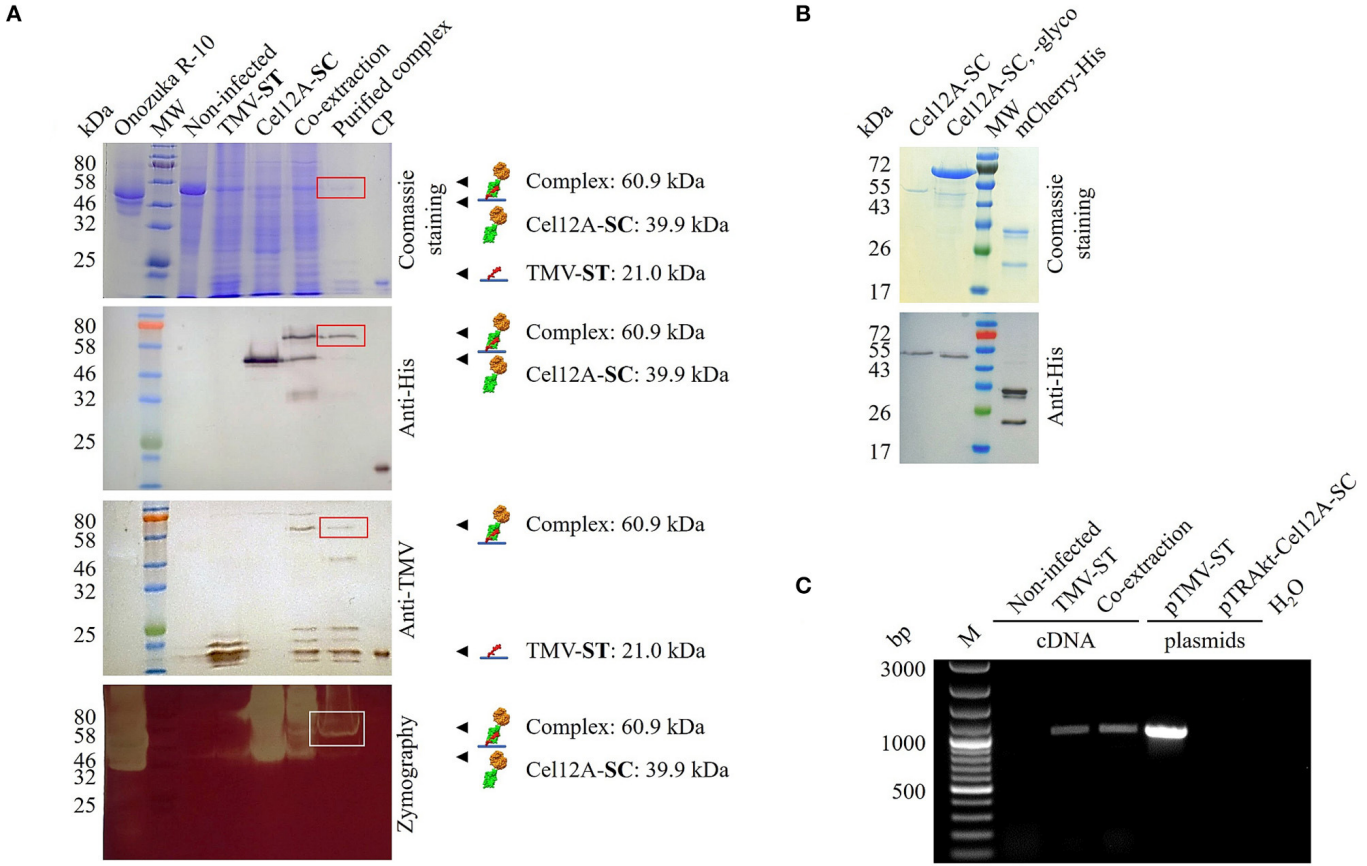
Analysis of purified TMV particles displaying Cel12A. **(A)** We separated 15 μl leaf extracts or 2.5 μg purified particles by SDS-PAGE followed by Coomassie Brilliant Blue staining or protein transfer to a nitrocellulose membrane for antibody detection with anti-His/GAM^AP^ and anti-TMV/GAR^AP^. Complex formation (boxes) was confirmed in co-extracts and purified complexes, and enzyme activity was confirmed by CMC zymography (50°C, 15 min). **(B)** We treated 1 μg of purified Cel12A-SC with endoglycosidase H (~70 kDa). **(C)** RT-PCR analysis of TMV-ST. RNA extracted from systemically infected and infiltrated leaves was reverse transcribed and amplified using TMV-specific primers. Plasmids pTMV-ST and pTRAkt-Cel12A-SC were used as controls. M, 100 bp+ DNA ladder; CP, *E. coli-*derived TMV CP containing a His_6_ tag; mCherry-His, purified control protein (27.5 kDa); MW, protein molecular weight ladder with **(A)** P7712, **(B)** P7719; Non-infected, noninfected *N. benthamiana* extract; Onozuka R-10, multicomponent enzyme system from *Trichoderma viridae*; SC, SpyCatcher; ST, SpyTag. Other lanes are extracts from leaves infected with TMV-ST or infiltrated with Cel12A-SC.

The expression of TMV-ST and Cel12A-SC, as well as complex formation in the co-extracts, was confirmed by SDS-PAGE and western blot analysis with specific antibodies, revealing the anticipated molecular weights of 21.0, 39.9, and 60.9 kDa (Figure 2A). Given the presence of two putative glycosylation sites, Cel12A-SC was detected as a ~50 kDa band in the western blot probed with the anti-His antibody, also showing a higher molecular weight for the complex. The treatment of Cel12A-SC with endoglycosidase H shifted the molecular weight of protein to ~45 kDa (Figure 2B). Purified TMV-ST/Cel12A-SC nanoparticles were detected as discrete signals with anti-His and anti-TMV antibodies (Figure 2A). The coupling efficiency was 46% as determined by densitometric analysis. The immobilized enzyme remained active against CMC as shown by zymography assay at 50°C (Figure 2A).

TMV-ST/Cel12A-SC nanoparticles were analyzed by RT-PCR to detect viral genomic RNA (Figure 2C). Total RNA was extracted from TMV-ST infected leaves or those mixed with the corresponding Cel12A-SC infiltrated leaves, and the encapsidated viral genomic RNA was reverse transcribed into cDNA and amplified using primers specific for TMV. The samples containing TMV-ST or TMV-ST co-extracted with Cel12A-SC yielded a band of ~991 bp as expected. No bands were visible in the control lanes (noninfected *N. benthamiana* leaf extracts and water). The plasmid controls generated fragments for pTMV-ST but not for pTRAkt-Cel12A-SC.

**Figure 3:**
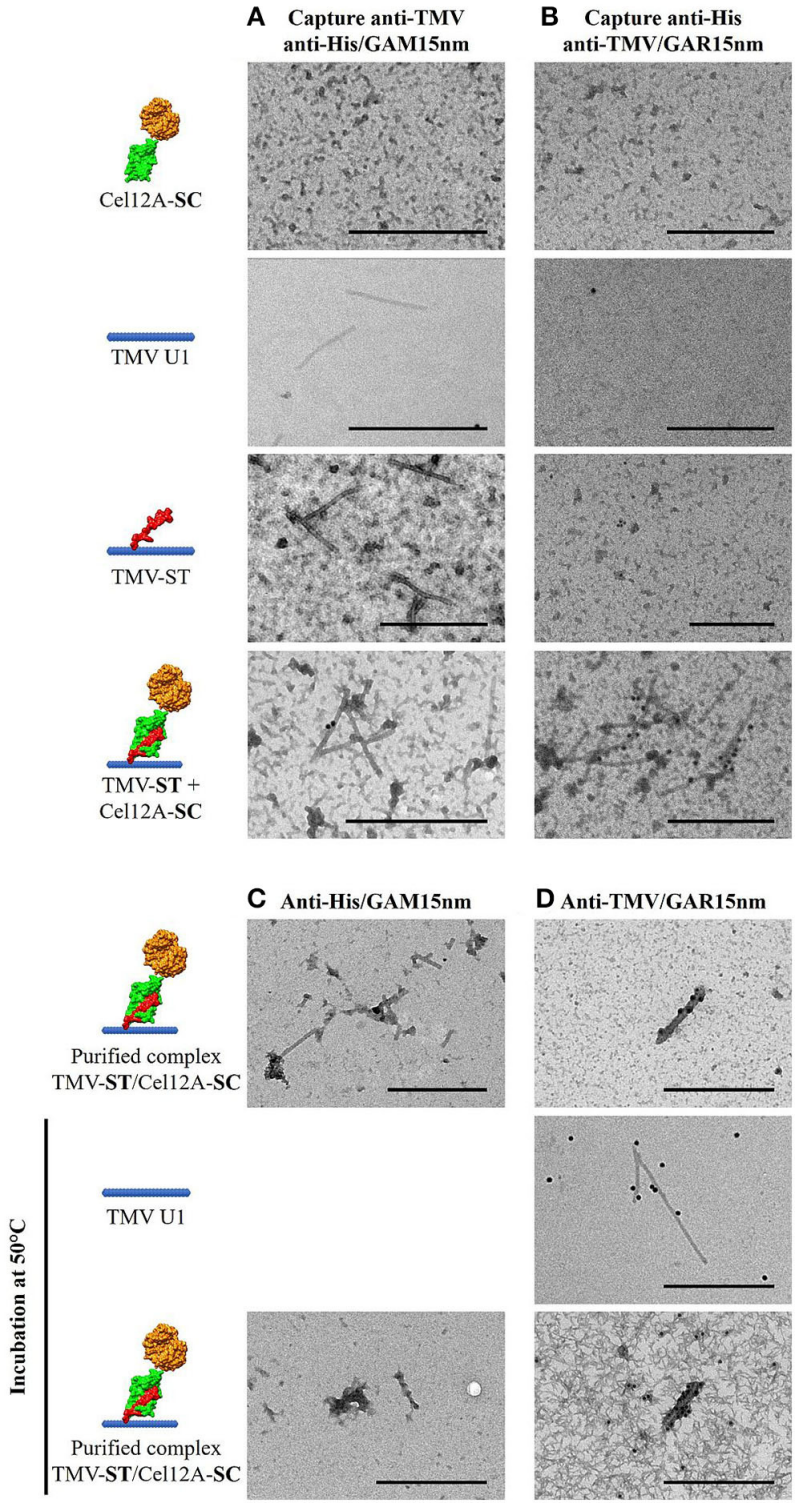
Transmission electron micrographs showing Cel12A covalently attached to modified TMV particles. **(A)** Particles were captured from leaf extracts using anti-TMV antibodies and the endoglucanase decorated with anti-His/GAM15nm gold conjugate, or **(B)** Cel12A was captured using anti-His antibodies and the particles were decorated with anti-TMV/GAR15nm gold conjugate. The purified TMV-ST/Cel12A-SC particles were decorated with the **(C)** anti-His/GAM15nm or **(D)** anti-TMV/GAR 15nm gold conjugates, with or without prior heat pretreatment at 50°C. Cel12A, *T. reesei* endoglucanase Cel12A; SC, SpyCatcher; ST, SpyTag. Scale bar = 500 nm.

The covalent attachment of Cel12A-SC to TMV-ST was also confirmed by immunosorbent electron microscopy (Figure 3). The nanoparticles and cellulases were captured from leaf extracts using anti-TMV and anti-His antibodies, respectively, and were decorated using the corresponding anti-His/GAM15nm or anti-TMV/GAR15nm gold conjugates. As anticipated, no particles were observed in the Cel12A-SC control with either antibody combination (Figures 3A,B). The wild-type TMV and TMV-ST particles were captured solely by the anti-TMV antibody but were not decorated with anti-His/GAM15nm gold particles (Figures 3A,B). Only co-extracts containing both TMV-ST and Cel12A-SC yielded gold-decorated particles with both capture/decoration methods (Figures 3A,B). The morphology of the purified TMV-ST/Cel12A-SC nanoparticles was similar to that of wild-type TMV (300 × 18 nm rigid rods) and the particles were labeled with anti-His/GAM15nm and anti-TMV/GAR15nm confirming the presence of the attached enzyme (Figures 3C,D). To confirm the particle stability at optimal temperatures for the endoglucanase, wild-type TMV and the purified TMV-ST/Cel12A-SC complex were incubated at 50°C for 1 h before probing with specific antibody-gold conjugates as above, revealing intact, gold-decorated particles (Figures 3C,D).

**Figure 4:**
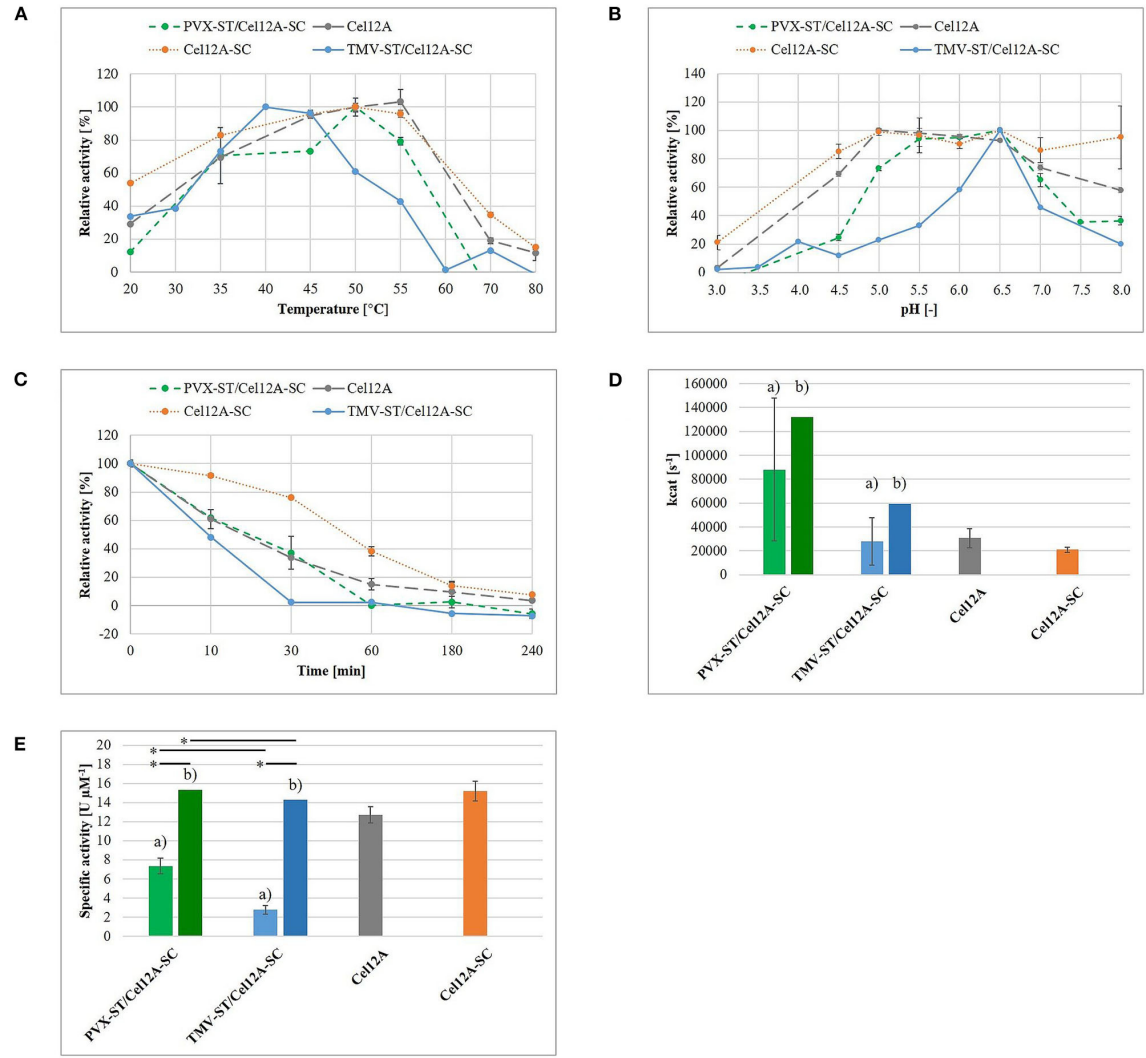
Enzymatic activity of TMV-ST/Cel12A-SC nanoparticles. The enzymatic activity of immobilized *T. reesei* Cel12A was measured using an Azo-CMC assay **(A–C,E)** or a 4-MUC assay **(D)**. **(A)** Endoglucanase activity in the temperature range 20–80°C. The maximum activity for each protein was set to 100%. **(B)** Endoglucanase activity in the pH range 3.0–8.0. The maximum activity for each protein was set to 100%. **(C)** Retention of enzymatic activity following preincubation at 50°C for 10–240 min at the optimal temperature and pH conditions for each protein. The enzymatic activity of each enzyme without preincubation was set to 100%. **(D)** Empirical (a) turnover number kcat calculated from Michaelis-Menten data at the optimal temperature and pH for each protein. Estimated kcat (b) is extrapolated and corresponds to a 100% enzyme coverage. **(E)** Empirical (a) and estimated (b) specific activity of free and immobilized Cel12A. Cel12A, *T. reesei* endoglucanase Cel12A; SC, SpyCatcher; ST, SpyTag. Data are means ± SD (*n* = 3). *p < 0.05. Adapted with permission (Röder et al., 2017b) 2017, WILEY-VCH Verlag GmbH & Co. KGaA.

The enzymatic activity of the purified TMV-ST/Cel12A-SC complex was investigated using Azo-CMC assays to define optimal catalysis conditions and 4-MUC assays for the analysis of enzyme kinetics, in comparison to purified endoglucanase fusions and PVX-ST/Cel12A-SC from our previous study (Figure 4) (Röder et al., 2017b). The relative catalytic activity of purified TMV-ST/Cel12A particles on Azo-CMC was tested in the temperature range 20–80°C and the pH range 3.0–8.0 (Figures 4A,B). Cel12A, Cel12A-SC fusion proteins and PVX-ST/Cel12A-SC complexes showed an optimum temperature range of 50–55°C, whereas the optimal temperature for Cel12A-SC immobilized on TMV-ST particles was lower (40–45°C). The optimum temperature for each configuration was set to 100% allowing the calculation of relative enzymatic activities for TMV-ST/Cel12A-SC nanoparticles at 20°C (33.7 ± 1.0%) and 55°C (42.8 ± 3.8%). In contrast to free Cel12A, free Cel12A-SC, and Cel12A-SC immobilized on PVX particles, no activity was detected at higher temperatures when we used TMV as the carrier.

Fusing the endoglucanase to SC not only increased the optimal pH from 5.0 to pH 6.5, but also increased the relative activity at acidic and neutral pH values. TMV-ST/Cel12A-SC displayed an optimal pH of 6.5, but compared to other Cel12A fusions, we observed no relative activity at pH 3.5 or pH 8.0, and only 12.0 ± 5.2% relative activity at pH 4.5, and 45.7 ± 8.8% at pH 7.0.

The retention of enzymatic activity was investigated using an Azo-CMC assay at the optimal temperature and pH for each enzyme, following preincubation at 50°C for 10–240 min (Figure 4C). The fusion of Cel12A (33.7 ± 0.6%) to SC (76.1 ± 0.9%) and its immobilization on PVX particles (37.2 ± 11.5%) achieved a ~2.2-fold higher residual activity following preincubation for 30 min (Röder et al., 2017b). In contrast, TMV-ST/Cel12A-SC particles retained only minimal relative activity (2.5 ± 1.0%).

The kinetic parameters were calculated for the substrate 4-MUC and fitted to the Michaelis-Menten equation (Figure 4D, Table 1). Compared to Cel12A-His, Cel12A-SC demonstrated a ~1.4-fold lower V_max_ and k_cat_, and a ~2.0-fold lower K_m_, whereas PVX-ST/Cel12A-SC achieved a ~2.9-fold higher V_max_ and k_cat_, but a ~3.5-fold higher K_m_. TMV-ST/Cel12A-SC particles were characterized by slightly lower V_max_ and k_cat_ values than the Cel12A control. The catalytic activity (k_cat_/K_m_) of Cel12A-SC was ~1.4-fold higher than the control, but the catalytic activity of PVX-ST/Cel12A-SC was ~1.2-fold lower than the control and that of TMV-ST/Cel12A-SC was ~2.4-fold lower than the control.

**Table 1:** Comparison of kinetic parameters for Cel12A fusion proteins and Cel12A immobilized on viral nanoparticles.

**Construct**	**V_**max**_ [M s^**−1**^]**	**K_**m**_ ± SD [M]**	**k_**cat**_ ± SD [s^**−1**^]**	**k_**cat**_/K_**m**_ [M^**−1**^ s^**−1**^]**
Cel12A (Röder et al., 2017b)	229.8	16.1 ± 5.2	30,641 ± 7908	1903.2
Cel12A-SC (Röder et al., 2017b)	158.6	8.0 ± 1.2	21,142 ± 2,179	2642.8
PVX-ST/Cel12A-SC^a^ (Röder et al., 2017b)	659.9	56.7 ± 41.3	87,992 ± 59,723	1551.9
PVX-ST/Cel12A-SC^b^ (Röder et al., 2017b)	659.9	56.7 ± 41.3	131,987 ± 89.6	2327.8
TMV-ST/Cel12A-SC^a^	208.0	34.8 ± 27.7	27,734 ± 19,866	797.1
TMV-ST/Cel12A-SC^b^	208.0	34.8 ± 27.7	59,431 ± 42,570	1708.1

a *Empirical values for ~67% (PVX) and 46% (TMV) coupling efficiency*.

b *Estimated values for 100% coupling efficiency*.

*Adapted with permission (Röder et al., 2017b) 2017, WILEY-VCH Verlag GmbH & Co. KGaA*.

The specific activity of the immobilized endoglucanases was determined using Azo-CMC as a model substrate, adjusted to 1 μM protein (Figure 4E). Cel12A and Cel12A-SC showed specific activities of 12.7 ± 0.8 and 15.2 ± 1.0 U μM^−1^, respectively. The specific activity of Cel12A was higher when attached to PVX (7.4 ± 0.8 U μM^−1^) than TMV (2.8 ± 0.4 U μM^−1^).

## Discussion

Enzyme immobilization can address many of the limitations of soluble enzymes, including low activity, stability, selectivity, and reusability (Liese and Hilterhaus, 2013; Sheldon and Woodley, 2018). This efficient technique was based on the multienzyme complexes produced by anaerobic cellulolytic bacteria, particularly the surface-displayed cellulosomes containing scaffoldings with different cohesin modules that bind with high affinity to complementary dockerin domains present in many cellulases (Artzi et al., 2017). An additional carbohydrate-binding module enables cellulases to access their substrates. This modular structure has been developed into artificial designer cellulosomes to facilitate the saccharification of lignocellulosic substrates, which is considered a major bottleneck in the cost-effective degradation of plant-derived biomass (Gunnoo et al., 2016). Although designer cellulosomes mimic the natural spatial organization of enzymes, they have yet to surpass the efficiency of their natural counterparts. Moreover, recombinant scaffolds are usually expressed from long open reading frames, which can produce unstable mRNAs with low expression levels (Walker and Vierstra, 2007). Alternative carrier materials have therefore been investigated, including polymeric, magnetic, and mesoporous scaffolds, and various nanomaterials, to enhance catalytic performance (Xie et al., 2009; Misson et al., 2015). The suitability of such materials depends on their robustness and the cost of immobilization.

Virus nanoparticles can be produced sustainably in large quantities by molecular farming, and have a precisely ordered three-dimensional structure (Rossmann, 2013). Cowpea mosaic virus, TMV, and PVX are frequently used as carriers for drugs, epitopes, peptides, and fluorescent markers, attached by chemical conjugation or genetic engineering (Culver et al., 2015; Lico et al., 2015; Wen and Steinmetz, 2016; Narayanan and Han, 2017; Röder et al., 2019). However, few publications thus far have discussed catalyst-decorated plant VNPs, probably reflecting the constraints affecting the size, pI, amino acid composition, and posttranslational modifications of displayed proteins added by genetic engineering (Carette et al., 2007; Aljabali et al., 2012; Pille et al., 2013; Cuenca et al., 2016). One of the first examples of plant VNP enzyme display used genetically engineered PVX to display *Candida antartica* lipase B (Carette et al., 2007). In this study, enzyme expression was facilitated by using the foot-and-mouth disease virus (FMDV) 2A ribosomal skip sequence, allowing the enzyme to be displayed on ~25% of the CP subunits. Enzyme activity was reduced ~45-fold compared to the free protein, and could be imaged by laser scanning confocal microscopy. An alternative approach based on the biotin–streptavidin system allowed glucose oxidase and horseradish peroxidase to be displayed on TMV particles and increased enzyme activity by up to 45-fold (Koch et al., 2015). The authors presented a two-step coupling method that was suitable for the preparation of multienzyme complexes (Conrado et al., 2008; Horn and Sticht, 2015), and decorated ~50% of the TMV CP subunits with the enzymes. Activity declined by ~50% after 2 weeks of storage, but 75% residual activity remained after eight catalytic cycles. TMV is therefore suitable as a carrier, with tight positional control and steric accessibility of enzymes combined with stability, regenerability, and reusability. However, the method was based on chemical conjugation, restricting it to reactive groups that do not impair enzyme functions, and requiring a large excess of the target molecule (Schlick et al., 2005; Holder et al., 2010; Venter et al., 2011). Recently, the authors reported the ST/SC covalent attachment approach as a cost-effective one-step surface display technique that can auto-immobilize proteins such as. *T. reesei* endoglucanase Cel12A on the surface of PVX and enhance its activity (Röder et al., 2017b).

Given the advantages of TMV as an enzyme carrier, we used the ST/SC system to streamline the enzyme immobilization process. A recombinant TMV vector was produced by fusing the *st* sequence to the 3′ end of the *cp* gene with additional sequences encoding a glycine-serine linker and a pI-adjusting peptide (Figure 1). The latter shifts the overall pI of the basic ST peptide fusion (pI = 9.6) to a value that enables systemic infection in *N. benthamiana* following mechanical inoculation. Endoglucanase Cel12A-SC and controls were transiently expressed in *N. benthamiana* by agroinfiltration as described in the previous study of the authors (Röder et al., 2017b). Genomic RNA was isolated from the recombinant particles and the presence of the *cp*–*st* sequence was confirmed by RT-PCR. TMV-ST and Cel12A-SC formed a stable complex in the co-extracts, as verified by western blot (Figure 2). The molecular weights of Cel12A-SC and TMV-ST/Cel12A-SC were higher than predicted because the endoglucanase was targeted to the endoplasmic reticulum, enabling glycosylation. The NetNGlyc 1.0 server (Technical University of Denmark) predicted two putative glycosylation sites at positions Asn166 and Asn 169 of Cel12A-SC, and an *N*-acetylglucosamine residue was previously shown to be attached to Asn164 of Cel12A (Sandgren et al., 2001).

Layered-bead chromatography was used to purify TMV-ST/Cel12A nanoparticles, indicating a coupling efficiency of ~46%, comparable to the biotin–streptavidin system (Koch et al., 2015). The affinity of ST/SC (K_D_ = 10^−10^ M) is lower than that of biotin/avidin (K_D_ = 10^−15^ M), although still very high, but the complex formation is reported to be ~20 times stronger (Zakeri et al., 2012), explaining the growing interest in ST/SC technology (Hatlem et al., 2019). TMV virus-like particles (VLPs) were recently engineered to attach three enzymes in *E. coli*, taking advantage of the substrate channeling effect to synthesize a precursor drug against malaria (Wei et al., 2020). The potential for steric hindrance must also be taken into account during enzyme immobilization. Complete occupancy was reported for tandem hepatitis B core VLPs attached to green fluorescent protein (GFP) or HIV capsid protein p24 *in vivo*, whereas a negative correlation between the size and density of displayed antigens was reported on *Acinetobacter* phage AP205 spherical VLPs (Thrane et al., 2016; Peyret et al., 2020). TMV is also able to build virus-like particles (VLPs). But due to the physiological pH, this is not possible *in planta* as shown in Peyret et al. (2020) for Hepatitis B core-like particles. For a successful assembly of TMV VLPs, the CP subunits have to be dialyzed against an acidic pH. This procedure results in less stable particles compared to the VNPs assembled in plants with virus RNA. Furthermore, the attached enzymes need a specific pH for optimal activity, which is pH 6.5 in case of Cel12A. At this pH value, TMV VLPs disassemble into disks again and the advantage of immobilization on a nanoparticle would be lost. We therefore excluded this approach from the present study and focused on TMV VNPs. The use of filamentous VNPs may help to overcome steric hindrance not only by providing a greater surface area but also a larger number of potential attachment sites.

Transmission electron microscopy confirmed the attachment of Cel12A-SC to TMV-ST particles (Figure 3). The endoglucanase was labeled with anti-His/GAM15nm immunogold only on TMV particles displaying ST, as expected. Engineered VNPs were also stable following incubation at 50°C for 1 h, a temperature at which Cel12A remains active, as shown by zymography (Figure 2). TMV therefore appears to be a suitable carrier for thermostable enzymes.

An empirical hydrolysis model with the substrates Azo-CMC and 4-MUC was used to determine the enzymatic activity of TMV particles decorated with Cel12A, as previously reported for Cel12A, Cel12A-SC, and PVX-ST/Cel12A-SC (Röder et al., 2017b). Figure 4 compares the data from the current and previous studies to highlight the differences in enzymatic activity and kinetic parameters when Cel12A-SC is immobilized on TMV-ST nanoparticles. The optimal pH was comparable for the immobilized and free enzyme (pH 6.5), but the optimal temperature of TMV-ST/Cel12A was 40°C, making it less thermotolerant than free Cel12A, free Cel12A-SC, or PVX-ST/Cel12A-SC. A striking decrease in relative enzymatic activity at higher temperatures and non-optimal pH values was also observed, thus making the TMV carrier unsuitable for industrial processes requiring high temperatures or extreme pH conditions (Figures 4A,B). Under optimal reaction conditions, TMV-ST/Cel12A-SC retained almost no activity toward Azo-CMC after incubation at 50°C for 30 min, whereas the counterpart PVX-ST/Cel12A-SC retained 37.2 ± 11.5% of its activity under these conditions (Figure 4C).

Different concentrations of 4-MUC were used to determine the kinetic parameters of the TMV-ST/Cel12A-SC particles, and the data were fitted to the Michaelis-Menten equation (Figures 4D,E, Table 1). The characteristics of the TMV scaffold strongly influenced the activity of the enzyme. The coupling efficiency of PVX-ST/Cel12A-SC was ~1.5-fold higher, and the flexible PVX scaffolds displayed ~850 enzymes (~67% coverage) whereas the rigid TMV scaffolds displayed ~980 enzymes (~46% coverage). Moreover, TMV-ST/Cel12A-SC particles adsorbed to the substrate ~1.6-fold faster than PVX-ST/Cel12A-SC particles, but were still not as fast as the control enzyme (~2.1-fold lower affinity). Substrate binding is usually the rate-limiting step in an enzymatic reaction, but Cel12A-SC converted ~3 times as much substrate to product per unit of time when PVX rather than TMV was the carrier, indicating significant steric hindrance by TMV. The rigid composition of TMV-ST, although equipped with an additional flexible linker, not only reduces the coupling efficiency but also the specific activity and catalytic efficiency by about 2-fold. This effect was still present even when the coupling efficiency was taken into account. We extrapolated the enzymatic activity based on the overall protein content, and compared it to the different templates and control enzymes. If all 2,130 TMV or all 1,270 PVX CP subunits would display endoglucanases, the PVX carrier still outperforms TMV and soluble Cel12A, and the PVX-ST/Cel12A-SC complex therefore remains the more efficient biocatalyst. This is probably explained by the positional control and helical arrangement of the CP subunits, which is advantageous for effective biomass degradation. One turn comprises 8.9 CP_PVX_ or 16.3 CP_TMV_ subunits, resulting in helical pitches of 34.5 and 22.9 Å, respectively (Parker et al., 2002; Ge and Zhou, 2011). The average distance between cellulases (e.g., in the *C. thermocellum* cellulosome) is ~40 Å, and this is an important determinant of catalytic activity (Mayer et al., 1987). Surprisingly, activity declines as the distance between enzymes closes, explaining why the activity of Cel12A was ~2.5-fold higher when immobilized on PVX rather than TMV particles, where more endoglucanase entities are concentrated on a shorter scaffold (Molinier et al., 2011; Vazana et al., 2013; Bae et al., 2015).

The search for new carrier materials suitable for enzyme immobilization has drawn attention to recombinant VNPs, which have robust, organized, and programmable structures, as well as straightforward and cost-effective production methods. Chemical conjugation was initially used to immobilize enzymes on VNPs because posttranslational modifications are often required for enzyme activity. However, traditional conjugation methods are time consuming, expensive, and need a large excess of coupling reagents, so researchers have turned their attention to the comparably much simpler ST/SC technology. Here, the authors showed that *T. reesei* endoglucanase Cel12A can be immobilized on TMV particles using the ST/SC system, as we previously reported for PVX. It was found that Cel12A activity increased when immobilized on PVX particles, but this was not the case for TMV even though the TMV particles displayed more Cel12A molecules than PVX. This suggests the immobilized enzymes were less active on this rigid scaffold, possibly due to steric limitations caused by the helical arrangement of CP subunits. Further work is required to increase the specific activity of Cel12A by adding synergistic enzymes to the same scaffold, thereby promoting substrate channeling. The stoichiometry should also be optimized to achieve effective biomass degradation. Enzymes needed in smaller amounts could be presented by the attachment of K-tagged fusion proteins using SpyLigase, or by using the FMDV 2A sequence to reduce the coupling efficiency (Fierer et al., 2014; Röder et al., 2017a). Although PVX outperforms TMV as a carrier for Cel12A, TMV has great potential for other applications due to the dense and stable presentation of target peptides on a highly organized and stable protein-based platform.

## Data Availability Statement

The raw data supporting the conclusions of this article will be made available by the authors, without undue reservation.

## Author Contributions

JS provided the idea of the work, designed and conducted the experiments, and wrote the paper. UC and JS participated in the interpretation of results and critically reviewed the manuscript. Both authors read and approved the final manuscript.

Vector sequences can be found in the Supplementary Material. Additional data, a more general and in-depth discussion can be found in the author's thesis (http://publications.rwth-aachen.de/record/732253/files/732253.pdf).

## Conflict of Interest

The authors declare that the research was conducted in the absence of any commercial or financial relationships that could be construed as a potential conflict of interest.

## Publisher's Note

All claims expressed in this article are solely those of the authors and do not necessarily represent those of their affiliated organizations, or those of the publisher, the editors and the reviewers. Any product that may be evaluated in this article, or claim that may be made by its manufacturer, is not guaranteed or endorsed by the publisher.

## Abbreviations

4-MUC: 4-methylumbelliferyl-β-d-cellobioside
CP: coat protein
CMC: carboxymethylcellulose
DD: pI-adjusting sequence encoding the peptide DEADDAED
dpi: days post-inoculation
G_4_S: peptide linker consisting of four glycines and one serine; repeated 3 times
pI: isoelectric point
PVX: Potato virus X
ROS: reactive oxygen species
SC: SpyCatcher
ST: SpyTag
TMV: Tobacco mosaic virus
VLPs: virus-like particles
VNPs: plant virus nanoparticles.
