# Supplementary material for: Analysis of Engineered Tobacco Mosaic Virus and Potato Virus X Nanoparticles as Carriers for Biocatalysts
Source: Front Plant Sci. 2021 Aug 6;12:710869. doi: 10.3389/fpls.2021.710869 (PMC8377429; doi:10.3389/fpls.2021.710869)
Supplement: Supplementary file 1 [file Data_Sheet_1.PDF]

frontiers

Supplementary Material

Analysis of Engineered Tobacco Mosaic Virus and Potato Virus X Nanoparticles as Carriers for Biocatalysts

Juliane Schuphan[1], Ulrich Commandeur[1*]

[1] Institute for Molecular Biotechnology, RWTH Aachen University, Worringerweg 1, 52074 Aachen, Germany

*** Correspondence:**
Prof. U. Commandeur
ulrich.commandeur@molbiotech.rwth-aachen.de

1 Nucleotide sequences of recombinant TMV-ST

pTMV-ST

The coat protein is indicated in black, the glycine-serine linker is underlined, blue indicates the DD pI-adjusting sequence, and red indicates SpyTag. The vector pTMV-ST is derived from pTRBO (Lindbo, 2007).

ATGCCTTATACAATCAACTCTCCGAGCCAATTTGTTTACTTAAGTTCCGCTTATGCAGAT
CCTGTGCAGCTGATCAATCTGTGTACAAATGCATTGGGTAACCAGTTTCAAACGCAACA
AGCTAGGACAACAGTCCAACAGCAATTTGCGGATGCCTGGAAACCTGTGCCTAGTATGA
CAGTGAGATTTCCTGCATCGGATTTCTATGTGTATAGATATAATTCGACGCTTGATCCGT
TGATCACGGCGTTATTAAATAGCTTCGATACTAGAAATAGAATAATAGAGGTTGATAAT
CAACCCGCACCGAATACTACTGAAATCGTTAACGCGACTCAGAGGGCAGACGATGCGAC
TGTAGCTATAAGGGCTTCAATCAATAATTTGGCTAATGAACTGGTTCGTGGAACTGGCAT
GTTCAATCAAGCAAGCTTTGAGACTGCTAGTGGACTTGTCTGGACCACAACTCCGGCTA
CT<u>GGAGGTGGAGGTAGCGGCGGTGGAGGGAGTGGTGGAGGCGGTAGC</u>GACGAGGCTGA
TGACGCAGAAGATATGGCTCATATTGTTATGGTTGATGCTTATAAGCCTACTAAGTAA

pTRAkt-Cel12A-SC

The leader peptide is indicated in blue, the *T. reesei* endoglucanase is indicated in orange, glycine-serine linker is underlined, red indicates the SpyCatcher, green indicates the His-Tag, and the retention signal for the endoplasmic reticulum in indicated in purple. The vector pTRAkt-Cel12A-SC is a derivate of the plant expression vector pPAM (GenBank: AY027531).

ATGGAGTGGAGCTGGATCTTCTTGTTCTTGCTCAGCGGCACTGCAGGTGTTCACTCCATG
TTTGCACAAACCAGCTGTGACCAGTGGGCAACCTTCACTGGCAACGGCTACACAGTCAG
CAACAACCTTTGGGGAGCATCAGCCGGCTCTGGATTTGGCTGCGTGACGGCGGTATCGC

TCAGCGGCGGGGCCTCCTGGCACGCAGACTGGCAGTGGTCCGGCGGCCAGAACAACGTC
AAGTCGTACCAGAACTCTCAGATTGCCATTCCCCAGAAGAGGACCGTCAACAGCATCAG
CAGCATGCCCACCACTGCCAGCTGGAGCTACAGCGGGAGCAACATCCGCGCTAATGTTG
CGTATGACTTGTTCACCGCAGCCAACCCGAATCATGTCACGTACTCGGGAGACTACGAA
CTCATGATCTGGCTTGGCAAATACGGCGATATTGGGCCGATTGGGTCCTCACAGGGAAC
AGTCAACGTCGGTGGCCAGAGCTGGACGCTCTACTATGGCTACAACGGAGCCATGCAAG
TCTATTCCTTTGTGGCCCAGACCAACACTACCAACTACAGCGGAGATGTCAAGAACTTCT
TCAATTATCTCCGAGACAATAAAGGATACAACGCTGCAGGCCAATATGTTCTTAGCTAC
CAATTTGGTACCGAGCCCTTCACGGGCAGTGGAACTCTGAACGTCGCATCCTGGACCGC
ATCTATCAACGGAGGTGGAGGTAGCGGCGGTGGAGGGAGTGGTGGAGGCGGTAGCGAT
TACGATATCCCGACGACGGAAAATCTGTATTTTCAAGGAGCCATGGTGGATACCTTATC
CGGACTGAGTAGTGAACAGGGTCAATCAGGAGACATGACAATTGAGGAAGATTCCGCA
ACGCACATTAAATTTAGTAAACGTGACGAGGACGGGAAAGAGCTGGCGGGTGCGACGA
TGGAGCTCCGTGACTCCAGTGGCAAAACTATTTCTACGTGGATCAGCGACGGACAGGTT
AAAGATTTTTACTTGTACCCGGGCAAATATACATTTGTGGAAACGGCGGCGCCGGACGG
ATACGAGGTGGCCACCGCGATCACCTTTACCGTCAATGAACAGGGCCAGGTGACTGTGA
ACGGCAAGGCCACCAAGGGGGATGCCCACATCGCGGCCGCTCATCACCATCACCATCAC
TCTGAGAAAGATGAGCTCTAG

2 References

Lindbo, J. A. (2007). TRBO: A high-efficiency tobacco mosaic virus RNA-based overexpression vector. *Plant Physiol.* 145, 1232–1240. doi:10.1104/pp.107.106377.
